# The C-terminal domain of T9SS component protein SprA assists *Flavobacterium psychrophilum* bacteriophage endolysin Ely174 to lyse Gram-negative bacteria

**DOI:** 10.1128/aem.01891-25

**Published:** 2025-10-22

**Authors:** Shuaishuai Xie, Yaoyajie Lu, Xueer Li, Yifan Xu, Xianglin Cao, Jianjun Chen

**Affiliations:** 1 College of Life Science, Henan Normal University 719561, Xinxiang, China; University of Milano-Bicocca, Milan, Italy

**Keywords:** endolysin, T9SS, thermal stability, protein engineering

## Abstract

*Flavobacterium psychrophilum* is the pathogen of bacterial cold-water disease, causing enormous economic losses in aquaculture. Antibiotic therapy to control *F. psychrophilum* risks the development of drug-resistant strains. The use of phage-derived endolysins as effective substitutes for antibiotics is a current research hotspot. In this study, the endolysin Ely174 of *F. psychrophilum* bacteriophage was heterologously expressed. The optical density of Triton-pretreated *F. psychrophilum* at 600 nm was decreased from 0.8 to 0.2 in approximately 6 min using 2.5 µg/mL endolysin Ely174. In addition to its wide pH tolerance range, Ely174 also displayed a broad host spectrum. The presence of Mg^2+^, Ca^2+^ and Na^+^ in the reaction system enhanced its bactericidal activity. The lytic activity of Ely174 was increased threefold by random mutagenesis. The thermal stability of cold-adapted Ely174 has been improved by rational design. The final superimposing mutation resulted in variant A39H/P48I/E144A, which lysed pretreated *F. psychrophilum* rapidly even after thermal treatment at 50°C for 2 h. The type IX secretion system (T9SS) is involved in the pathogenic mechanisms of bacteria in the phylum *Bacteroidetes*. The fusion of endolysin Ely174 with the C-terminal domain of the T9SS component protein SprA enabled Ely174-CTD_SprA_ to lyse untreated *F. psychrophilum*. Recombination with ABC transport permease could help endolysin Ely174 overcome the outer membrane barrier of Gram-negative bacteria. These results indicate the potential of endolysin Ely174 as an antibacterial agent in aquaculture and the food industry. Protein engineering of the endolysin Ely174 also provides a new perspective for the control of T9SS-containing bacteria.

**IMPORTANCE:**

*Flavobacterium psychrophilum* is a major pathogenic bacterium of salmonids. The antibiotic treatments used to control bacterial diseases in aquaculture are associated with food safety and environmental pollution issues. The application of phage-derived endolysins is a promising approach to replace antibiotics and prevent the emergence of drug-resistant bacteria. In this study, *F. psychrophilum* bacteriophage endolysin Ely174 exhibited bactericidal activity in different environments, including extreme pH, bovine serum, and cations. The lytic activity and thermal stability of endolysin Ely174 were improved by a combination of random mutagenesis and rational design. The protein engineering of endolysin Ely174 with a type IX secretion system element or functional protein enabled Ely174 to enter the peptidoglycan layer of Gram-negative bacteria and exert its function in the absence of outer membrane permeabilizers. The study findings expand our understanding of the control of T9SS-containing pathogens and provide more possibilities for further engineering modifications of endolysins.

## INTRODUCTION

The genus *Flavobacterium* is widespread in soil, water, and air. Several species are fish pathogens, such as *Flavobacterium psychrophilum* and *Flavobacterium columnare*. The Gram-negative bacterium *F. psychrophilum* is a main pathogen of salmonids. Bacterial cold-water disease (BCWD) in rainbow trout caused by *F. psychrophilum* infection typically results in tail erosion, dorsal fin injury, spleen enlargement, enteritis, and ascites (1, 2). The mortality rate owing to devastating *F. psychrophilum* infections in rainbow trout fry is as high as 90%. Ayu sweetfish, sea lamprey, and other non-salmonid freshwater fish are also affected by *F. psychrophilum* (3, 4). Vertical and horizontal transmission of *F. psychrophilum* increases the prevalence of BCWD (5). Economic losses caused by *F. psychrophilum* in the aquaculture industry should not be ignored. The majority of antibiotics that are effective in controlling BCWD outbreaks are not permitted for use in the food industry. In addition, the heavy use of antibiotics has led to the emergence of drug-resistant *F. psychrophilum*. Environmental pollution and food safety issues caused by antibiotic residues are a global concern (6). Therefore, there is an urgent need to identify effective methods other than antibiotics to prevent and control the transmission of *F. psychrophilum*.

Bacteriophages and their derivatives are currently being researched as potential alternatives to antibiotics (7, 8). Endolysins encoded by bacteriophages lyse host bacterial cells to release progeny phages. The peptidoglycan (PG) layer, a highly conserved component of the cell wall of Gram-positive bacteria and cell envelope of Gram-negative bacteria, is the primary site of endolysin hydrolysis. The unique targeting mechanism of endolysins makes them a promising novel and effective alternative antibacterial agent to deal with drug-resistant bacteria (9). Several endolysins for Gram-positive bacteria have entered the clinical trial stage because of their high lytic efficiency and lack of risk of bacterial resistance (10, 11). However, the presence of an outer membrane (OM) protects the PG layer of Gram-negative bacteria from exogenous endolysin attack. In addition to the search for endolysins with OM permeability to counteract the proliferation of Gram-negative bacteria. Combinations with citric acid, chloroform, Triton, ethylenediaminetetraacetic acid (EDTA), and other OM permeabilizers (OMPs), fusion with a transmembrane peptide or cationic peptide, or encapsulation in materials with penetrating properties are commonly used to improve the OM permeability of endolysins targeting Gram-negative bacteria (12, 13). The successful overcoming of the OM barrier will result in an expansion of the range of applications of endolysins.

Effective inhibition of Gram-negative bacteria usually requires high doses of endolysin (14). Most free endolysins are sensitive to external environmental factors, such as ion concentration, serum, and pH. A significant number of endolysins are prone to inactivation at high temperatures (12). Therefore, the practical applications of endolysins are impeded by the challenges inherent to formulation, storage, and transportation. Researchers have attempted to identify more effective ways to engineer endolysins and improve their properties. Multiple sequence alignments are commonly used to identify the conserved regions of endolysins and the key residue sites involved in their lytic activity (15). Based on three-dimensional structure analysis, molecular dynamics simulations, and calculations, the amino acid sites of endolysin were predicted and mutated to achieve the desired enzyme properties by rational design (16). For instance, the lytic activity and thermal stability of lysin PlyAB1 are promoted by point mutations (17). Previous studies have mainly focused on the molecular modification of endolysins against *Salmonella*, *Escherichia coli*, *Acinetobacter baumannii*, and other common food and clinical pathogenic bacteria. However, the modification of endolysins to combat bacterial diseases in aquaculture has rarely been reported.

The type IX secretion system (T9SS) is present only in members of the phylum *Bacteroidetes*. A minimum of 24 proteins are involved in the function of T9SS (18). The trans-periplasmic space motor complex is composed of GldK, GldL, GldM, and GldN (19). SprA forms a large β-barrel on the OM that serves as a T9SS cargo protein-conducting channel (20). Other components of the T9SS participate in the regulation, processing, and modification of cargo proteins. The conserved C-terminal domain of most substrates targets them to the T9SS translocon (21). T9SS and its cargo proteins play a crucial role in the pathogenesis of pathogenic bacteria (22). The T9SS of *F. psychrophilum* is responsible for virulence, adhesion, biofilm formation, and the secretion of proteins for gliding or biomacromolecular degradation (23). Endolysins fused with the membrane-translocating domains of bacteria can increase OM permeability (9). However, the combination of T9SS-derived elements and endolysins has not yet been studied. The binding of bacteriocin to endolysin has also been successful in controlling pathogens in the absence of OMPs (24). The recombination of endolysins with other functional proteins to improve their characteristics is yet to be fully developed.

In this study, the *F. psychrophilum* phage endolysin Ely174 was expressed. Its bactericidal effects and enzymatic properties have also been characterized. In addition, the lytic activity and thermal stability of Ely174 were improved by a combination of random mutation and rational design. Furthermore, co-expression with functional protein or fusion with a T9SS element allowed the engineered endolysin Ely174 to exert its bactericidal activity against Gram-negative bacteria without the aid of OMP. These findings contribute to the development of endolysins for controlling pathogens in intensive aquaculture.

## RESULTS

### Expression and lytic activity of endolysin Ely174

**Fig 1:**
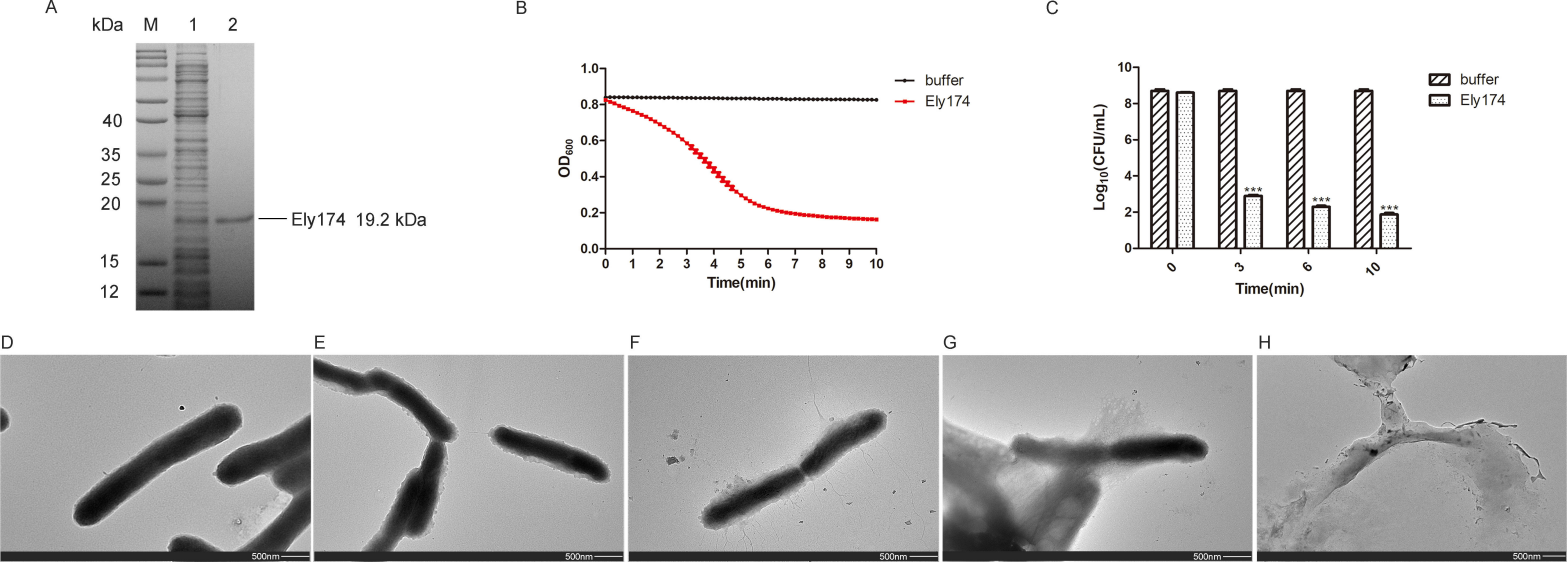
Lytic activity of endolysin Ely174. (**A**) SDS-PAGE profiles of endolysin Ely174. Lane M, molecular mass marker; Lane 1, soluble proteins of endolysin Ely174 expression strain; Lane 2, purified endolysin Ely174. (**B**) The bactericidal activity of endolysin Ely174 was measured using the turbidity method. Ely174 or Tris-HCl buffer was added to the Triton-pretreated *F. psychrophilum* cells. (**C**) The bactericidal activity of endolysin Ely174 at varying action times was assayed through CFU counts. (***P < 0.001). (**D–H**) Representative transmission electron micrograph images of *F. psychrophilum* treated by Tris-HCl buffer (**D**), Triton (**E**), and endolysin Ely174 (**F–H**).

Phages and their derivatives have great potential for the control of devastating fish pathogens (25). The genomic information of *F. psychrophilum* bacteriophage 6H was published in 2013 (26). However, the endolysin associated with its ability to lyse *F. psychrophilum* has not been thoroughly studied. Endolysin of phage 6H is composed of 174 amino acids, thus it was designated as endolysin Ely174. A recombinant plasmid containing the encoding region of Ely174 was expressed in *Escherichia coli* BL21. After the induction of expression and purification, a protein band corresponding to the molecular weight of endolysin Ely174 (19.2 kDa) was obtained (Fig. 1A; Fig. S1).

Due to the impermeability of the OM, most exogenous endolysins require the assistance of OMPs for the effective elimination of Gram-negative bacteria. The bactericidal activity of endolysin Ely174 was evaluated using the turbidity method (27). *F. psychrophilum* JC isolated from diseased rainbow trout in China was used for detection. The optical density at 600 nm (OD_600_) of *F. psychrophilum* pretreated with Triton decreased from 0.85 to 0.2 in 6 min with the addition of 2.5 µg/mL endolysin Ely174 (Fig. 1B). When the same volume of Tris-HCl buffer was added to the control group, there was no significant change in the OD_600_ value of Triton-pretreated *F. psychrophilum*. Colony-forming unit (CFU) counts showed that Triton-pretreated *F. psychrophilum* was reduced to 3.0 log following incubation with endolysin Ely174 for 3 min. Furthermore, Ely174 was able to kill Triton-pretreated *F. psychrophilum* by 6.3 log after 10 min of reaction (Fig. 1C). The results indicate that endolysin Ely174 is effective in eliminating Triton-pretreated *F. psychrophilum*.

To detect the lytic ability of endolysin Ely174, transmission electron microscopy (TEM) was used to observe the altered morphology of *F. psychrophilum*. The cell surface of natural *F. psychrophilum* was complete and smooth (Fig. 1D). After Triton treatment, the *F. psychrophilum* profile was blurred, but the bacteria remained intact (Fig. 1E). However, in the presence of endolysin Ely174, *F. psychrophilum* cells appeared loose, deformed, and broken (Fig. 1F and G). The released cellular contents and cell debris were clearly visible in observation fields (Fig. 1G and H). These results indicate the highly efficient activity of Ely174 in lysing Triton-pretreated *F. psychrophilum*.

### Optimum temperature and pH for endolysin Ely174 activity

**Fig 2:**
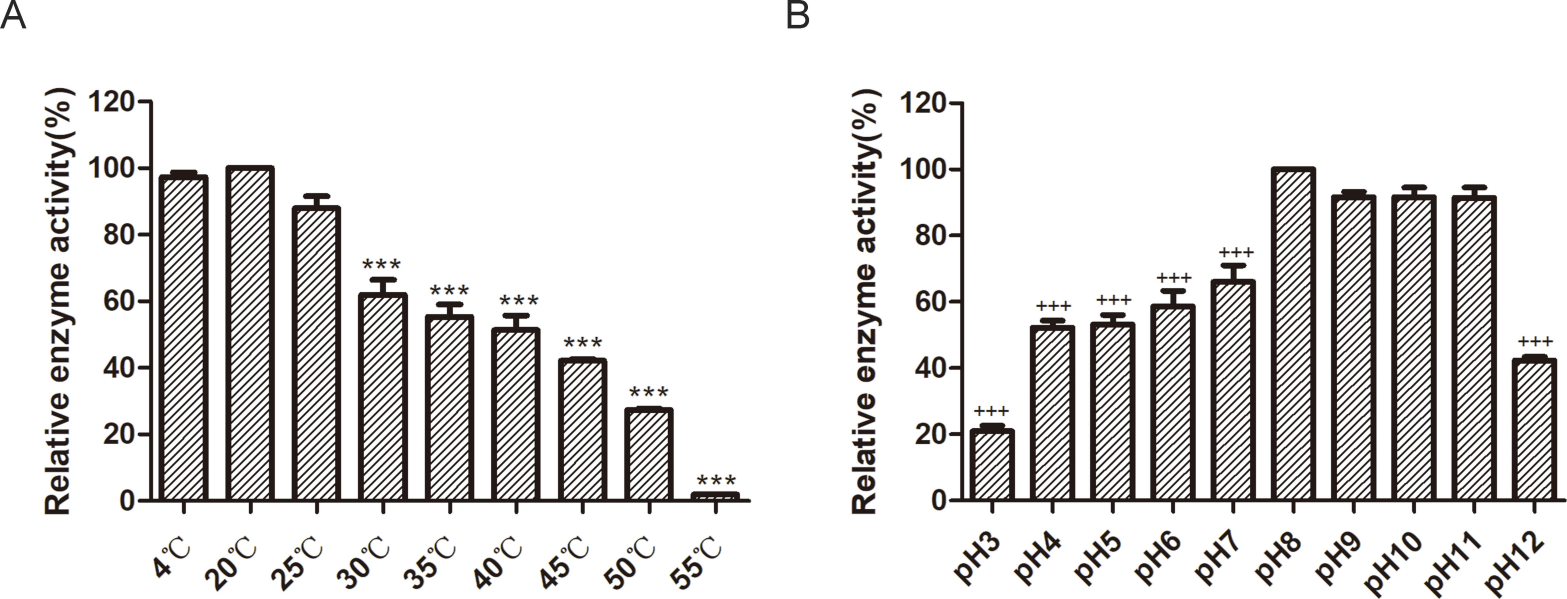
Optimal temperature (**A**) and pH (**B**) for the lytic activity of endolysin Ely174. Triton-pretreated *F. psychrophilum* was used for the detection. (***P < 0.001 VS. 4°C, ^+++^*P* < 0.001 vs pH 8).

Cold-adapted enzymes have been and continue to be identified in psychrophilic species (28). Given the nature of proteinases, it was necessary to determine the optimal temperature for Ely174 activity. Tests conducted at temperatures ranging from 4°C to 55°C revealed that Ely174 displayed the highest lytic activity at 20°C (Fig. 2A). Temperatures between 30°C and 40°C had some deleterious effect on the activity of endolysin Ely174, with the relative enzyme activity decreasing to approximately 50%. The bactericidal activity of endolysin Ely174 was almost completely lost at 55°C. However, endolysin Ely174 was active at pH 3.0 to 12.0, with an optimal pH for antibacterial activity of 8.0. The relative enzyme activity remained above 90% at pH 9.0 to 11.0, whereas the lytic activity decreased by approximately 50% at pH 4.0 to 7.0 (Fig. 2B). The results suggest that the enzymatic activity of endolysin Ely174 is enhanced under alkaline conditions.

### Effects of cations and serum on endolysin Ely174 lytic activity

**Fig 3:**
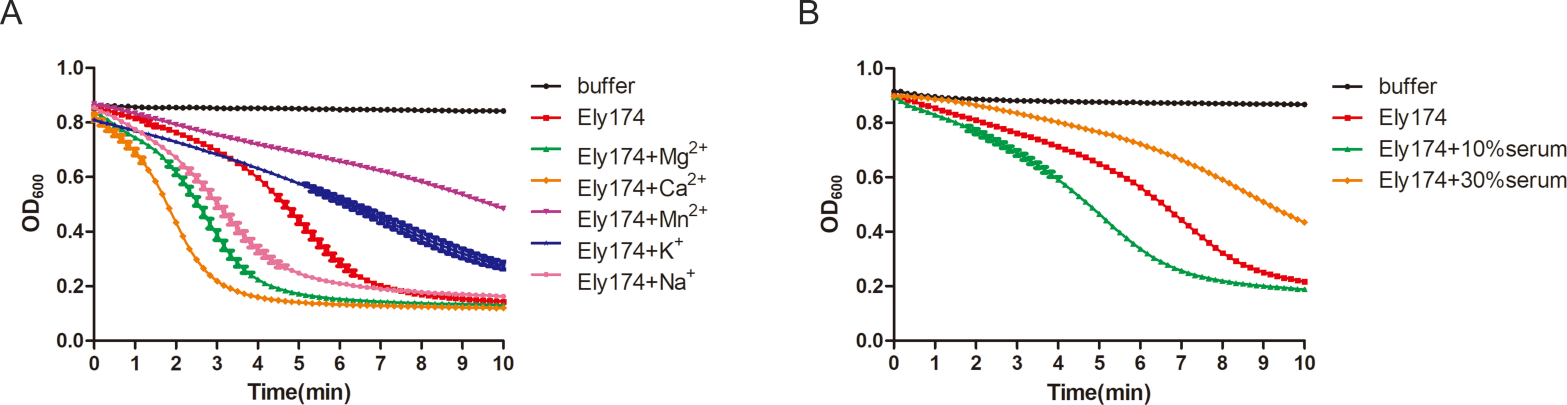
The lytic activity of endolysin Ely174 is affected by cations and serum. Different cations (**A**) or different concentrations of serum (**B**) were added to the reaction systems of Ely174. The final concentrations of endolysin Ely174 were 2.5 µg/mL (**A**) and 2.0 µg/mL (**B**) for the lysis of Triton-pretreated *F. psychrophilum*.

Lipopolysaccharides are negatively charged components of the OM that play a crucial role in the stability of Gram-negative bacteria. Positive ions in the reaction system affect the stability of host bacterial OM and the bactericidal activity of endolysin. The lytic efficiency of endolysin XFII against *E. coli* is reduced by cations (27). Accordingly, various cations were added to the endolysin Ely174 reaction buffer to test whether enzyme activity was affected. The addition of Mg^2+^, Ca^2+^, and Na^+^ enhanced the antibacterial efficiency of endolysin Ely174 compared with the reaction system lacking these cations. The enzymatic activity of endolysin Ely174 increased by at least twofold in the presence of Ca^2+^. K^+^ and Mn^2+^ decreased the ability of endolysin Ely174 to lyse *F. psychrophilum* (Fig. 3A). These results indicate that endolysin Ely174 is sensitive to the positive ions in the reaction system. The skin of fish infected with *F. psychrophilum* was always ulcerated. Serum has been identified as the principal cause of inactivation of endolysins under physiological conditions (9). The lytic activity of endolysin Ely174 increased by approximately 20% when 10% bovine serum was added to the reaction buffer. The addition of 30% bovine serum led to the inhibition of the enzyme activity of Ely174 (Fig. 3B). The results show that endolysin Ely174 can tolerate low concentrations of bovine serum.

### Host spectrum of endolysin Ely174

The similarities of the PG layers of Gram-negative bacteria may be one reason for the broad host spectrum of a number of endolysins (9). *F. psychrophilum* DSM 3660, *F. amnicola*, *F. bernardetii*, *F. geliluteum*, *Chryseobacterium piscicola*, *C. balustinum*, *C. oncorhynchi*, and other 11 aquatic bacteria were used to assess the bactericidal ability of endolysin Ely174 (Table 1). Thirteen Triton-pretreated strains were efficiently lysed by endolysin Ely174. Furthermore, the rapid decline in cell concentration showed that *F. facile* and *C. balustinum* were more sensitive to Ely174 (Fig. S3). In contrast, the antibacterial efficiency against the fish pathogen *Aeromonas hydrophila* was relatively low. These results indicate the broad lytic spectrum of endolysin Ely174.

**TABLE 1:** Lysis efficiency of endolysin Ely174 against different bacteria

Species	Strain	Lysis efficiency^*a*^
*Flavobacterium psychrophilum*	JC	+ + + +
*Flavobacterium psychrophilum*	DSM 3660	+ + +
*Flavobacterium flabelliforme*	P4023	+ + + +
*Flavobacterium geliluteum*	P7388	+ + + +
*Flavobacterium aquidurense*	DSM 18293	+ + + +
*Flavobacterium bernardetii*	F-372	+ + + +
*Flavobacterium amnicola*	LLJ-11	+ + + +
*Flavobacterium facile*	T-12	+ + + +
*Chryseobacterium oncorhynchi*	701B-08	+ +
*Chryseobacterium pennae*	1_F178	+ +
*Chryseobacterium scophthaimum*	LMG 13028	+ + +
*Chryseobacterium piscicola*	VQ-6316s	+ + + +
*Chryseobacterium balustinum*	NBRC 15053	+ + + +
*Chryseobacterium timonianum*	G972	+ +
*Chryseobacterium aquaticum*	10-46	+ + +
*Chryseobacterium pennipullorum*	7_F195	+ + +
*Aeromonas hydrophila*	MX16A	+
*Paeniglutamicibacter antarcticus*	SPC26	–

^*a*^ Lysis efficiency 0%–10%, –; lysis efficiency 11%–30%, +; lysis efficiency 31%–50%, + +; lysis efficiency 51%–70%, + + +; lysis efficiency 71% –100%, + + + +.

### Random mutation of endolysin Ely174 increases its lytic activity

**Fig 4:**
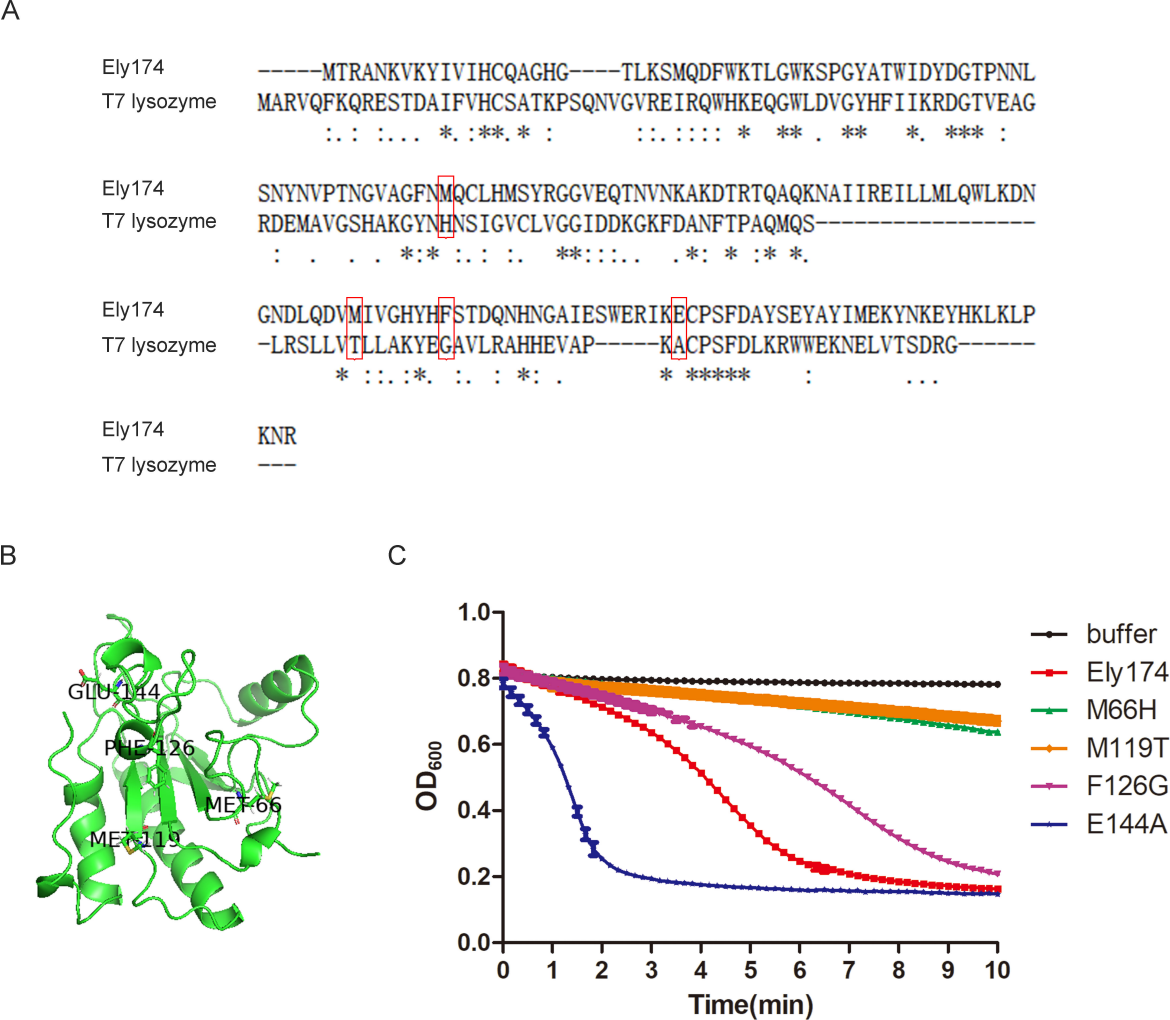
Random mutation increases the lytic activity of endolysin Ely174. (**A**) Sequence alignment of endolysin Ely174 and T7 lysozyme. (**B**) The predicted protein structure of endolysin Ely174. The amino acid residues selected for mutation are labeled. (**C**) The lysis efficiency of endolysin Ely174 and its mutants for Triton-pretreated *F. psychrophilum*.

AlphaFold was used to gain further insight into the characteristics of endolysin Ely174. The protein structure of T7 lysozyme was employed as a template for the homologous modeling of Ely174 (Fig. 4B). T7 lysozyme, encoded by *Escherichia* phage, belongs to the N-acetylmuramoyl-L-alanine amidase family. The sequence alignment of T7 lysozyme and Ely174 revealed a certain degree of similarity in their conserved regions (Fig. 4A). Random mutations are the basis for constructing mutant libraries and are indispensable for identifying proteins with specific traits in directed molecular evolution (29). Amino acid residues Met66, Met119, Phe126, and Glu144 of endolysin Ely174 were selected at random for mutation analysis (Fig. 4A and B). Altering Met66 or Met119 severely reduced the antibacterial efficacy of endolysin Ely174. Among the four variants, only E144A exhibited a significant increase in lytic activity. The enzymatic activity of the variant E144A was threefold higher than that of endolysin Ely174 (Fig. 4C). The results suggest that random mutagenesis is one way to enhance the killing efficiency of endolysin Ely174.

### Improved thermal stability of endolysin Ely174

**Fig 5:**
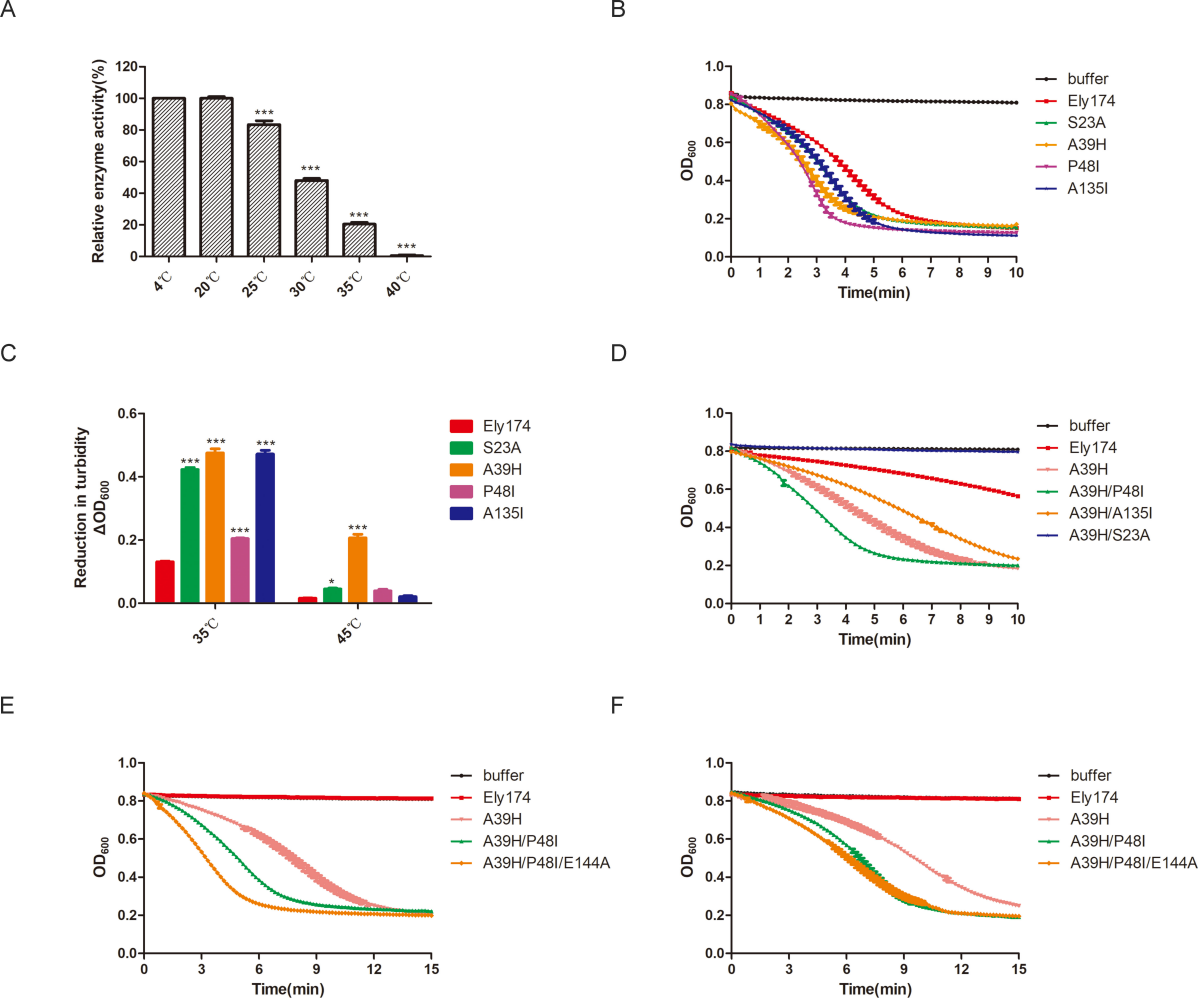
Improving the thermal stability and enzyme activity of endolysin Ely174 through rational design. (**A**) Thermal stability of endolysin Ely174. The enzymatic activity of endolysin Ely174 was assessed after incubation at different temperatures for 2 h. (***P < 0.001). (**B**) Lytic activity of single-point variants of Ely174 at 20°C. (**C**) Bactericidal activity of single-point variants of Ely174 after pretreatment at 35°C or 45°C for 2 h. (*P < 0.05). (**D**) Enzymatic activity of double-point variants of Ely174 after pretreatment at 35°C for 2 h. (**E–F**) Lytic activity of triple-point variants of Ely174 after pretreatment at 45°C (**E**) or 50°C (**F**) for 2 h. The detections were performed using Triton-pretreated *F. psychrophilum* cells.

Thermal stability of endolysins is important for their processing and applications (30). The lytic activity of Ely174 was measured after treatment at different temperatures for 2 h. The typical temperatures of the aquaculture environment are below 30°C. It was observed that the relative enzyme activity of endolysin Ely174 was maintained at 45% when the pretreatment temperature was 30°C. Treatment at 35°C for 2 h reduced the antibacterial activity to 20% (Fig. 5A). Therefore, it is necessary to improve the thermal stability of endolysin Ely174 to expand its application.

The simulation structure of endolysin Ely174 was analyzed using HotSpot Wizard 3.0. Based on sequence consensus and amino acid frequency, variants of endolysin Ely174 with potentially improved thermal stability were designed (Table 2). The lytic activities of the 10 single-point mutants were analyzed. In comparison with endolysin Ely174, the bactericidal activities of variants S23A, A39H, P48I, and A135I were increased at 20°C (Fig. 5B). The lytic activities of the other mutants were equal to or lower than that of endolysin Ely174 (Fig. S4). In addition, after pretreatment at 35°C for 2 h, the relative enzyme activities of mutants S23A, A39H, P48I, and A135I increased 3.2-, 3.6-, 1.5-, and 3.6-fold, respectively, compared with that of endolysin Ely174. Notably, the thermal stability of mutant A39H was significantly improved. The lytic activity of A39H was most effective among the other variants after being pretreated at 45°C for 2 h (Fig. 5C). To further improve the thermal stability of Ely174, two-site combined mutagenesis was performed. As shown in Fig. 5D, the lytic activity of the double-point variant A39H/P48I increased by 45% compared with that of A39H after pretreatment at 35°C for 2 h. However, the enzymatic activity of mutant A39H/A135I was not superior to that of A39H. The S23A/A39H mutant completely lost bactericidal activity under the same conditions. Mutations at these two sites may affect the spatial conformation of endolysin.

**TABLE 2:** Variants of endolysin Ely174 designed by HotSpot Wizard 3.0

Amino acid residue	Variant(s)
Ser23	S23A / S23E
Ala39	A39H
Pro48	P48I
Ala62	A62K / A62R
Met71	M71I
His132	H132G
Asn133	N133D
Ala135	A135I

Variant E144A, with significant lytic activity, was obtained through random mutagenesis. To increase the lytic activity of Ely174 variants at high temperatures, a superimposed mutation was conducted. Compared with mutant A39H/P48I, the triple-point variant A39H/P48I/E144A displayed increased bactericidal activity after treatment at 45°C or 50°C for 2 h (Fig. 5E and F). In particular, the lytic activity of variant A39H/A135I/E144A was 1.5-fold higher than that of A39H/P48I at 45°C pretreatment condition. The results indicate that a combination of random mutations and rational design effectively improves the thermal stability of endolysin Ely174. Variant A39H/P48I/E144A with enhanced lytic activity and thermal stability, thereby increasing its potential for future applications.

### Protein engineering of endolysin Ely174 improves its OM permeability

**Fig 6:**
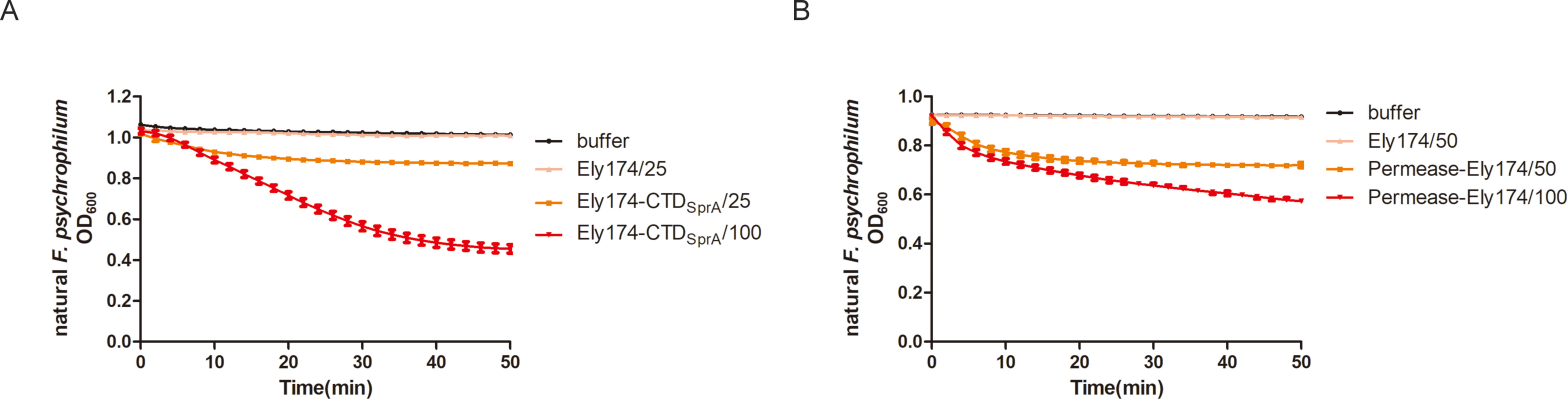
Lytic activity of the engineered endolysin Ely174. The bactericidal activities of Ely174-CTD_SprA_ (**A**) and Permease-Ely174 (**B**) were assayed using natural *F. psychrophilum* cells. Concentrations used for detections: Ely174-CTD_SprA_, 25 and 100 µg/mL; Permease-Ely174, 50 and 100 µg/mL.

The OM of Gram-negative bacteria limits the function of most endolysins without membrane-passing capacities (31). Peptides targeting the OM receptor can successfully deliver endolysins to the PG layer of host bacteria (12). T9SS is a recently discovered protein secretion system, and SprA forms a critical channel on the OM of T9SS-containing bacteria (32). However, the combination of T9SS and endolysin has not yet been reported. To address this, the C-terminal domain of SprA was fused with endolysin Ely174 to verify whether the T9SS element could increase the OM permeability of exogenous endolysin. As shown in Fig. 6A, in the absence of Triton pretreatment, the engineered endolysin Ely174-CTD_SprA_ was able to lyse natural *F. psychrophilum* cells. The OD_600_ of *F. psychrophilum* decreased from 1.00 to 0.40 in 50 min after the addition of 100 µg/mL Ely174-CTD_SprA_. Although the lytic rate of Ely174-CTD_SprA_ was slow, the results indicated that the C-terminal domain of SprA assisted endolysin in entering the PG layer of *F. psychrophilum* to exert its bactericidal function.

The ABC transporter permease has the capacity to transport or bind proteins (33). WP_011964122 is one of the conserved ABC transporter permeases in *F. psychrophilum*. When endolysin Ely174 was linked to the C-terminus of WP_011964122, the recombinant endolysin Permease-Ely174 reduced the OD_600_ of Triton-untreated *F. psychrophilum* cells from 0.90 to 0.60 within 50 min (Fig. 6B). Permease WP_011964122 is important for endolysin Ely174 to overcome the OM barrier of *F. psychrophilum*. The two recombinant endolysins exhibited relatively weak bactericidal ability at low concentrations. These results indicate that the OM permeability of endolysin Ely174 can be improved by protein engineering.

## DISCUSSION

Bacterial infections are one of the main causes of disease outbreaks in aquaculture (34). The first strain of *F. psychrophilum* was isolated from rainbow trout in 1948. Subsequently, it has been found in different fish species, including salmonids and non-salmonids (35). Progress on the development of the commercial vaccine targeting BCWD is slow (36). Developing antibacterial agents based on the mechanism of phages lysing host bacteria can help to reduce the overuse of antibiotics. An increasing number of phages with the ability to lyse *F. psychrophilum* have been identified (37). In contrast to phages, endolysins exert lytic activity immediately upon contact with the PG layer, making it impossible for host bacteria to accumulate mutations. In addition, endolysins control bacteria differently than antibiotics, thereby avoiding the risk of drug resistance (38). In this study, the enzymatic properties of *F. psychrophilum* bacteriophage endolysin Ely174 were explored.

The OM shields the PG layer of Gram-negative bacteria from external substances. One common method by which exogenous endolysins exert their enzymatic activity against Gram-negative bacteria is in combination with OMPs (30). Different concentrations of EDTA are usually employed to destroy the OM of bacteria (39). Triton could destabilize the OM of *A. baumannii*, thereby enabling PlyAB1 to exert its lytic activity (17). In the presence of endolysin Ely174, the optical concentration of Triton-pretreated *F. psychrophilum* decreased to the lowest detectable level within a short time. The damaged cells and leaked cell contents of *F. psychrophilum* further demonstrated the bactericidal ability of endolysin Ely174 (Fig. 1). The six images of *F. psychrophilum* treated with Ely174 showed that the number of lysed cells (152) exceeded that of intact cells (63) (Fig. S2). Approximately 70% of the *F. psychrophilum* cells were observed to be undergoing lysis. Despite the fact that a proportion of *F. psychrophilum* maintained structural integrity in the presence of Ely174, it is possible that a small number of cells may be in a viable but uncultivable state. Protein engineering is another effective method for improving the OM permeability of endolysin. Polycationic, hydrophobic, amphipathic peptides with membrane-penetrating properties are often used to deliver endolysins to the PG layer of host bacteria (12). For example, the fusion of cationic peptides to the C terminus of Lysep3 lysin could facilitate the lysis of *E. coli* without the co-administration of OMPs (40). When a peptide containing eight arginine residues was linked to the N-terminal of endolysin Ely174, in the absence of OMP, the natural *F. psychrophilum* was killed by the recombinant endolysin 8R-Ely174 (Fig. S6). The transport system and OM receptor of bacteria have also been employed as membrane-permeable targets for endolysins. Colicin-Lysep3 contacts the PG layer of *E. coli* via the TolB machinery (41). OM protein SprA forms a key channel for the translocation of T9SS cargo proteins and the acquisition of ions in the phylum *Bacteroidetes* (42). The C-terminal domain of SprA assisted endolysin Ely174 in lysing non-pretreated *F. psychrophilum* (Fig. 6A). It is worth highlighting that other T9SS-containing pathogens can be controlled in a similar manner. In comparison with the lytic ability of OMP-assisted Ely174, the recombinant endolysins Ely174-CTD_SprA_ required higher concentrations to kill natural *F. psychrophilum* (Fig. S7). It was also found that a high concentration (100 µg/mL) of the recombinant endolysin pesticin-T4 was required to penetrate the OM of *E. coli*. Furthermore, a considerable proportion of engineered endolysins demands a relatively prolonged action time to attain the desired level of killing efficiency (12). Other T9SS elements or suitable regions of SprA should be selected to improve the antibacterial efficiency of engineered endolysins in the further studies. ABC transporter systems are associated with bacterial virulence (43). The recombinant endolysin Permease-Ely174 lysed *F. psychrophilum* in the absence of Triton pretreatment (Fig. 6B). A previous study reported that the virulence factor aspartyl proteinases in *Candida albicans* are effectively inhibited by the binding of the ABC transporter permease protein from *Microcoleus chthonoplastes* (44). The exploration of other proteins with transport functions in *F. psychrophilum* may provide additional pathways for exogenous endolysins to penetrate the bacterial OM. The limited lytic activities of Ely174-CTD_SprA_ and Permease-Ely174 preliminarily indicated that the protein secretion and transport systems of *F. psychrophilum* could be used to engineer Ely174 to enhance its OM permeability.

The lytic activity of endolysins is highly sensitive to the external environment, owing to their proteinaceous nature. Notably, endolysin Ely174 showed superior antimicrobial activity under alkaline conditions (Fig. 2). The broad pH tolerance of Ely174 endolysin facilitates its potential for industrial production and practical application. The bactericidal activity of Ely174 was enhanced in the presence of Mg^2+^, Ca^2+^, and Na^+^ within the reaction system (Fig. 3A). Previous studies have reported that T9SS is required for the acquisition and assimilation of divalent cations Ca^2+^ and Mg^2+^ in *Cytophaga hutchinsonii* (42, 45). The lytic activity of endolysin MMPphg was activated by Mg^2+^ (46). However, Mg^2+^ and Ca^2+^ in the reaction buffer reduced the antibacterial efficiency of LysSE24 (47). The enzymatic activities of various endolysins are inhibited by serum in clinical applications. For example, the lytic activities of PlyA and PlyPa91 are completely lost in human serum (14, 48). The property of endolysin Ely174 adapted serum is significant for future *in vivo* bactericidal applications. The host spectrum of most endolysins is broad due to the conservation and similarity of the PG layer in different Gram-negative bacteria (49). Most bacteria inhibited by endolysin Ely174 belonged to the genera *Flavobacterium* and *Chryseobacterium* (Table 1). A common feature of these bacteria is the presence of the T9SS. T9SS may be a useful drug target for the development of endolysins to control T9SS-containing pathogens.

Random mutagenesis is used to screen for mutants with desirable properties (50). The lytic activity of endolysin Ely174 was successfully increased by random mutation (Fig. 4). However, the mutant library constructed by random mutagenesis was small and the acquisition rate of the target mutants was low. The majority of psychrophilic enzymes exhibit catalytic activity at low temperatures (51). As with other cold-adapted enzymes, endolysin Ely174 is influenced by the reaction temperature and thermal treatment (Fig. 2A and 5A). The processing and transportation of endolysin in practical applications have high requirements for thermal stability. Rational design is based on the sequence and structure of the protein, which in turn alters the nature of the enzyme. Thus, variants with target properties are more readily available (28, 52). Among the mutations designed by HotSpot Wizard 3.0, the thermal stability of four mutants was improved compared with endolysin Ely174. Finally, mutant A39H/P48I/E144A was obtained, exhibiting superior thermal stability and lytic activity (Fig. 5E and F). The heat stability of PlyAB1 was increased by superimposing mutations (17). The union of rational design and random mutagenesis effectively improved the efficacy of endolysin Ely174. The storage stability of endolysin is crucial for the commercialization of the product (9). In the absence of any protective agent, endolysin Ely174 remained active for approximately 75 days at 4°C (Fig. S5). However, the efficacy of Ely174 may be diminished due to extreme dilution in expansive aquatic environments. It must be acknowledged that Ely174 faces a range of practical challenges, including but not limited to delivery efficiency, host-microbial interaction, and *in vivo* half-life.

Our study not only characterized the properties of endolysin Ely174 but also improved its lytic activity and thermal stability. Endolysin Ely174 exerts bactericidal effects in complex environments, offering enhanced potential for controlling fish pathogens. The protein engineering of Ely174 to enhance its OM permeability may provide insights for the elimination of other pathogens containing the T9SS. Further research is needed to realize the application of Ely174 as an antimicrobial agent in the fishing and food industries.

## MATERIALS AND METHODS

### Bacterial strains and general growth conditions

The bacterial strains for this study are listed in Table S1. *E. coli* BL21 (DE3) was cultured in Luria-Bertani medium at 37°C or 16°C. *F. psychrophilum* JC and the other strains were cultured in tryptone yeast extract salts medium at 20°C (53). The concentration of kanamycin used in this study was 50 µg/mL.

### Construction of expression strain

The protein sequence of endolysin Ely174 was obtained from https://www.ncbi.nlm.nih.gov/protein/YP_008320451.1. Primers 6HF and 6HR were used to amplify the gene of endolysin Ely174 from the synthetic plasmid pQLL (Table S2). The amplified fragment containing His-tag was ligated to vector pET-29b using the HindIII and SalI sites to produce the expression vector pET-29b-endolysin. Finally, the recombinant vector was transformed into *E. coli* BL21 (DE3). Plasmids of the transformants were sequenced using T7 primers to screen for the required expression strains.

### Purification of Ely174

The expression strain was cultured to an OD_600_ of 0.6 at 37°C with shaking at 180 rpm. Isopropyl-β-D-thiogalactopyranoside was then added to the medium at a final concentration of 1 mM. Endolysin Ely174 was expressed at 16℃ with shaking at 90 rpm for 20 h, after which cells were collected by centrifugation at 4℃ for 10 min. The cells were then suspended in Tris-HCl buffer (20 mM, pH 7.4) for ultrasonic disruption. The resulting supernatant was collected following centrifugation at 10,000×*g* for 20 min at 4°C. Ely174 in the supernatant was further purified using an Ni-NTA column (Sangon Biotech, Shanghai). PBS buffer containing 50, 100, and 400 mM imidazole was used for elution. The concentration of proteins was measured by the Bradford method (54). SDS-PAGE was then used to visualize the purified proteins.

### Determination of Ely174 lytic activity

*F. psychrophilum* JC and the other strains used to assess the enzymatic activity of Ely174 were cultured to an OD_600_ of 0.6. The cells in the 100 mL culture were harvested and washed before being incubated with 1 mL of OMP buffer (1% Triton, 20 mM HEPES, 140 mM NaCl, pH 7.4) for 10 min. The cells were then washed twice, after which the concentration of the pretreated cells was adjusted to an OD_600_ of approximately 0.85 using Tris-HCl buffer. Finally, either 1 µL purified endolysin Ely174 (final concentration 2.5 µg/mL) or 1 µL Tris-HCl buffer was added to the prepared testing cells (199 µL) (55). The optical density of the reaction system was detected by a BioTek microplate reader (shaking 10 s, interval 10 s). The change of OD_600_ of the mixture was detected at 20°C. A total of three independent biological replicates were conducted. CFU counts were performed as previously reported (27). Triton-pretreated *F. psychrophilum* cells were incubated with endolysin Ely174 for 0, 3, 6, and 10 min. The colonies on the plate were counted after 7 d at 20°C.

### Observation by transmission electron microscopy

*F. psychrophilum* JC cells were pretreated with Triton as previously described. Subsequently, the cells were washed and adjusted to an OD_600_ of approximately 0.85. The cells were then incubated with endolysin Ely174 (2.5 µg/mL) at 20°C with gentle shaking for 2 min. Ely174 was removed by centrifugation, after which the *F. psychrophilum* cells were washed twice with PBS buffer. Glutaraldehyde (2.5%) was used to fix Triton-pretreated, Ely174-treated, or natural *F. psychrophilum* cells for 3 h. The washed cells were stained with phosphotungstic acid for 3 s. An FTI Tecnai G2 F20 transmission electron microscope was used to observe the cells (27). Three independent replicates were performed.

### Different reaction systems of Ely174

To assay the optimal temperature for the lytic activity of endolysin Ely174, Triton-pretreated *F. psychrophilum* cells were washed, and then the cells with an OD_600_ of 0.85 were mixed with purified Ely174 (2.5 µg/mL) and incubated at 4°C to 45°C. Triton-pretreated *F. psychrophilum* cells were suspended in Tris-HCl buffer at pH ranging from 3.0 to 12.0 to determine the optimum pH for the bactericidal activity of Ely174. The ΔOD_600_ values of the different reaction systems were calculated as initial OD_600_–OD_600_ of the reaction system at 3 min. The relative enzyme activity (%) was calculated as (ΔOD_600_ of each reaction system/ΔOD_600_ of the control group) × 100. Bovine serum (Sangon Biotech, Shanghai) or different cations were added to the Tris-HCl buffer used in the reaction system to detect the lytic activity of endolysin Ely174. The proportion of bovine serum used in the reactions was 10% and 30%. The final concentration of Mg^2+^, Ca^2+^, Mn^2+^, Na^+^, and K^+^ was 1 mM. For the lytic spectrum of Ely174, lysis efficiency (%) was calculated as [(OD_600_ of the reaction system at 0 min–OD_600_ of the reaction system at 8 min)/OD_600_ of the control at 0 min] × 100. Unless stated otherwise, the majority of the determinations were conducted at 20°C.

### Bioinformatics analysis of Ely174

Sequence alignment of endolysin Ely174 and T7 lysozyme was performed using CLUSTALW. The amino acid sequence of endolysin Ely174 was submitted to AlphaFold and Robetta for protein structure homology modeling. The variants of endolysin Ely174 were designed using HotSpot Wizard 3.0.

### Site-directed mutagenesis of Ely174

The amino acids of Ely174 were individually mutated to the desired ones. Primers 6HF and E144AR (containing the mutation site) were used to amplify the first half fragment of Ely174. The other half of Ely174 was amplified using the primers E144AF and 6HR. The complete fragment of Ely174 containing the mutation site E144A was obtained by overlapping PCR (56). Other variants of Ely174 were constructed using the same method.

### Thermal stability and storage stability of Ely174 and its variants

Purified endolysin Ely174 and its variants were incubated at different temperatures for 2 h. The lytic activity of the pretreated endolysins was then assayed at 20℃. For the thermal stability of the variants, reduction in turbidity ΔOD_600_ was calculated as initial OD_600_–OD_600_ of the reaction system at 6 min. The purified endolysin Ely174 was stored at 4°C, and its enzyme activity was determined at regular times. To assess the storage stability of endolysin, the reduction in turbidity ΔOD_600_ after 15 min was determined. All experiments were carried out using three biological replicates.

### Construction of engineered Ely174

The genome of *F. psychrophilum* DSM 3660 was used as a template. The C-terminal domain of SprA was amplified using the primers SprAF and SprAR. The final fragment containing endolysin Ely174 and CTD_SprA_ was obtained by the primers 6HF and SprAR by overlapping PCR (57). Fragment of *F. psychrophilum* permease was obtained using the primers ABCF and ABCR. The cells of *F. psychrophilum* JC were harvested, washed, and then suspended in Tris-HCl buffer for detection. The same volume of recombinant endolysin Ely174 or Tris-HCl buffer was added to the prepared cells. The BioTek microplate reader was used to record the antibacterial efficiency of the recombinant endolysin Ely174. Different concentrations of engineered endolysin Ely174 were used for detection.

### Statistical analysis

The statistical analysis was conducted using GraphPad Prism. The mean values and standard deviations of the data from three biological replicates were calculated, and error bars were generated accordingly. The CFU counts of Ely174 were analyzed using a *t*-test. The optimal temperature and pH, as well as the thermal stability of Ely174 and its variants, were analyzed using a one-way analysis of variance. Dunnett’s test was employed for the post-hoc test methodology. The lytic activity of Ely174 at 4°C or pH 8 was designated as the control. All columns compared with the control column. The statistical significance level was set at *P* < 0.05.

## SUPPLEMENTAL MATERIAL

Supplemental material aem.01891-25-s0001.docx Tables S1 and S2; Fig. S1 to S7.

ASM does not own the copyrights to Supplemental Material that may be linked to, or accessed through, an article. The authors have granted ASM a non-exclusive, world-wide license to publish the Supplemental Material files. Please contact the corresponding author directly for reuse.
