# Supplementary material for: The C-terminal domain of T9SS component protein SprA assists Flavobacterium psychrophilum bacteriophage endolysin Ely174 to lyse Gram-negative bacteria
Source: Appl Environ Microbiol. 2025 Oct 22;91(11):e01891-25. doi: 10.1128/aem.01891-25 (PMC12628812; doi:10.1128/aem.01891-25)
Supplement: Supplemental material — Tables S1 and S2; Fig. S1 to S7. [file aem.01891-25-s0001.docx]

**Title**

The C-terminal domain of T9SS component protein SprA assists *Flavobacterium psychrophilum* bacteriophage endolysin Ely174 to lyse Gram-negative bacteria

**Running title**

Protein engineering of endolysin Ely174

**Authors**

Shuaishuai Xie, Yaoyajie Lu, Xueer Li, Yifan Xu, Xianglin Cao, Jianjun Chen^#^

**Author’s affiliation**

College of Life Science, Henan Normal University, Xinxiang, 453007, China

# Address Correspondence to: Jianjun Chen, [chenjianjun@htu.edu.cn](mailto:chenjianjun@htu.edu.cn)

**Supplemental Material**

**Table S1** Strains and plasmids used in this study

| Strains or plasmids | Description | Source |
| --- | --- | --- |
|  | | |
| *E. coli* BL21 | Strain used for heterologous expression | TaKaRa |
| *Flavobacterium psychrophilum* | JC | Laboratory |
| *Flavobacterium psychrophilum* | DSM 3660 | Biobw |
|  |  |  |
|  |  |  |
|  |  |  |
|  |  |  |
|  |  |  |
| *Flavobacterium flabelliforme* | P4023 | Laboratory |
| *Flavobacterium geliluteum* | P7388 | Laboratory |
| *Flavobacterium aquidurense* | DSM 18293 | Laboratory |
| *Flavobacterium bernardetii* | F-372 | Laboratory |
| *Flavobacterium amnicola* | LLJ-11 | Laboratory |
| *Flavobacterium facile* | T-12 | Laboratory |
| *Chryseobacterium oncorhynchi* | 701B-08 | Laboratory |
| *Chryseobacterium pennae* | 1_F178 | Laboratory |
| *Chryseobacterium piscicola* | VQ-6316s | Laboratory |
| *Chryseobacterium balustinum* | NBRC 15053 | Laboratory |
| *Chryseobacterium timonianum* | G972 | Laboratory |
| *Chryseobacterium aquaticum* | 10-46 | Laboratory |
| *Chryseobacterium scophthaimum* | LMG 13028 | Laboratory |
| *Chryseobacterium pennipullorum* | 7_F195 | Laboratory |
| *Paeniglutamicibacter antarcticus* | SPC26 | Laboratory |
| *Aeromonas hydrophila* | MX16A | Laboratory |
| pET29b(+) | Plasmid for recombination protein construction | TaKaRa |
| pQLL | Plasmid containing the gene sequence of Ely174 | Sangon |
|  |  |  |
|  |  |  |
|  |  |  |

**Table S2** Sequences of the primers used in this study

| Primer | Sequence |
| --- | --- |
|  |  |
|  |  |
| 6H-F | GCGCGAGCTCATGACCAGAGCAAATAAAGTAAAGTAC |
| 6H-R | GCGCAAGCTTCTAATGGTGATGGTGATGATGTCTATT |
| T7 | TAATACGACTCACTATAGGG |
| T7-T | TGCTAGTTATTGCTCAGCGG |
| S23A-F | AACTCTAAAGGCCATGCAGGATTTTTGGAAAACACTT |
| S23A-R | TTTTCCAAAAATCCTGCATGGCCTTTAGAGTTCCGTG |
| S23E-F | ACGGAACTCTAAAGGAGATGCAGGATTTTTGGAAAAC |
| S23E-R | TCCAAAAATCCTGCATCTCCTTTAGAGTTCCGTGCCC |
| 39A-HF | ACTTGGTTGGAAATCTCCAGGTTATCATACTTGGATA |
| 39A-HR | ATCTATCCAAGTATGATAACCTGGAGATTTCCAACCA |
| 39R-D | ATCCAAGTATGATAACCTGGAGATTTCCAACCAAGT |
| P48I-F | AGATTATGATGGTACGATAAATAATCTTTCAAATTAT |
| P48I-R | TTGAAAGATTATTTATCGTACCATCATAATCTATCCA |
|  |  |
| F481 | ACTTGGATAGATTATGATGGTACGATCAATAATCTT |
| F482 | TAGATTATGATGGTACGATCAATAATCTTTCAAATT |
| A62K-F | AAATGGCGTAAAAGGATTTAATATGCAATGTTTACAT |
| A62K-R | ATTGCATATTAAATCCTTTTACGCCATTTGTAGGGAC |
| A62R-F | CCTACAAATGGCGTAAGGGGATTTAATATGCAATGTT |
| A62R-R | TGCATATTAAATCCCCTTACGCCATTTGTAGGGACAT |
| M66H-F | CGTAGCGGGATTTAATCAGCAATGTTTACATATGTCA |
| M66H-R | TATGTAAACATTGCTGATTAAATCCCGCTACGCCATT |
| M71I-F | AATGTTTACATATATCATACCGTGGTGGTGTAGAACA |
| M71I-R | ACCACCACGGTATGATATATGTAAACATTGCATATTA |
| M119T-F | AATGATTTACAAGATGTGACGATTGTGGGGCATTATC |
| M119T-R | CCCCACAATCGTCACATCTTGTAAATCATTTCCGTTG |
| F126G-F | GATTGTGGGGCATTATCATTTTTCTACAGATCAAAAT |
| F126G-R | TTTTGATCTGTAGAAAAATGATAATGCCCCACAATCA |
| H132G-F | CATTTTTCTACAGATCAAAATGGAAATGGGGCAATAG |
| H132G-R | TCTATTGCCCCATTTCCATTTTGATCTGTAGAAAAAT |
| N133D-F | ATCACGATGGGGCAATAGAGTCATGGGAACGTATAAA |
| N133D-R | CCATCGTGATTTTGATCTGTAGAAAAATGATAATGCC |
| A135I-F | ATGGGATAATAGAGTCATGGGAACGTATAAAAGAATG |
| A135I-R | CTATTATCCCATTGTGATTTTGATCTGTAGAAAAATG |
| 144E-AF | CATGGGAACGTATAAAAGCATGCCCTTCGTTCGATGC |
| 144E-AR | GCATCGAACGAAGGGCATGCTTTTATACGTTCCCATG |
| ABCF | GCGCGAGCTCATGCTAGGATTATTTAAAGAAAATGTA |
| ABCR | TTTGCTCTGGTCATCTCGTACCGCAAGGCTTCAATAG |
| ABC6HF2 | TTGCGGTACGAGATGACCAGAGCAAATAAAGTAAAGT |
| ABC6HR2 | GCGCGTCGACCTAATGGTGATGGTGATGATGTCTATT |
| 6HSprAR | TCCTAGTCCAATTCTATTTTTTGGAAGTTTCAATTTG |
| SprAF | CCAAAAAATAGAATTGGACTAGGATATCGTATAAAAG |
| SprAR | CGCGAAGCTTGTTTCCAAAATTATATCTAATTGTAAA |
| 8RF1 | CGCGAGCTCCGTCGCCGACGGAGACGTCGCCGAATGA |
| 8RF2 | CCGACGGAGACGTCGCCGAATGACCAGAGCAAATAAA |

^a^Restriction sites are underlined.


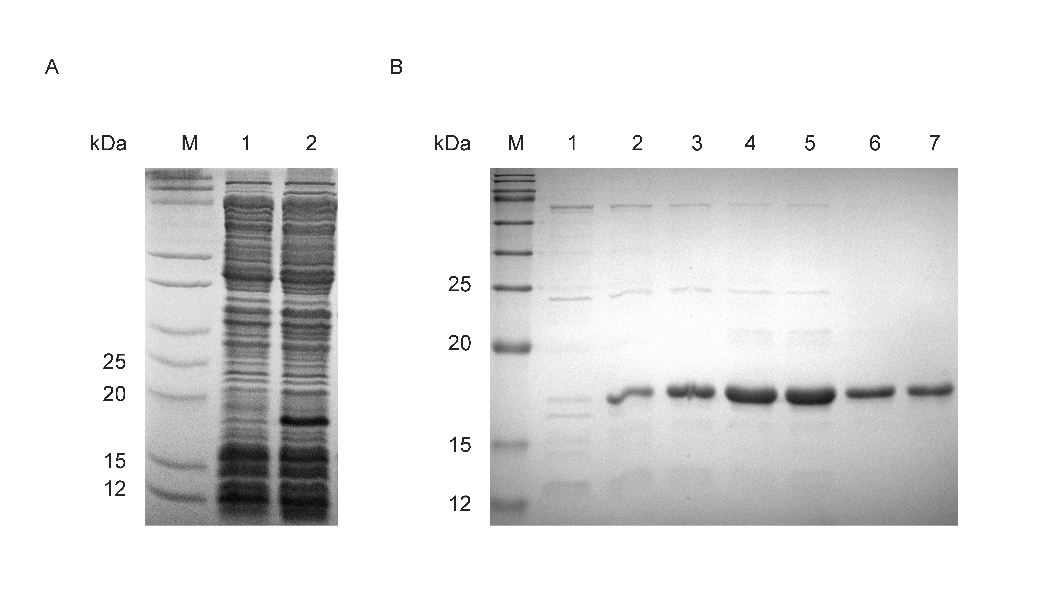


**FIG S1** Expression and purification of endolysin Ely174. (A) Soluble proteins of endolysin Ely174 expressing strain prior to (lane 1) and following (lane 2) induction. (B) Purification of endolysin Ely174. Endolysin Ely174 under elution conditions with different concentrations of imidazole. Lane 1 – 7: 50, 100, 150, 200, 250, 300, and 400 mM, respectively. Lane M: protein markers.


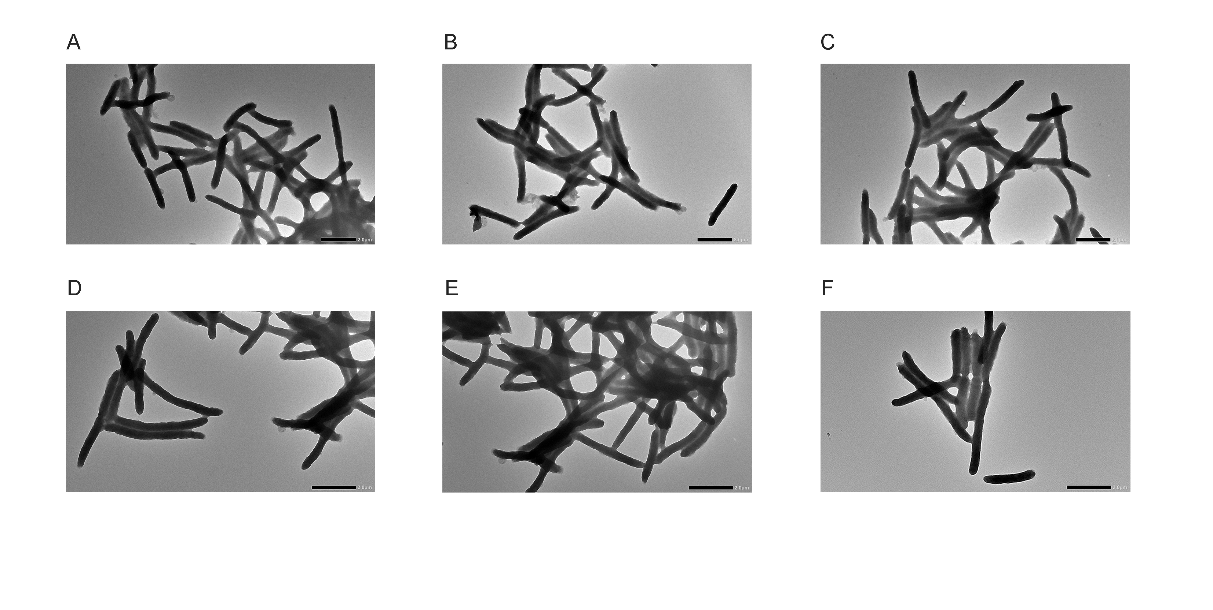


**FIG S2** Images of *F. psychrophilum* treated with Ely174 for 2 min (A-F). The relatively dispersed cells within the images were used for counting. A: 13 intact cells / 34 lysed cells ( lysed cells: 72.3%); B: 11 intact cells / 26 lysed cells (lysed cells: 70.2%); C: 17 intact cells / 37 lysed cells (lysed cells: 68.5%); D: 11 intact cells / 23 lysed cells (lysed cells: 67.6%); E: 7 intact cells / 22 lysed cells (lysed cells: 75.8%); F: 4 intact cells /10 lysed cells (lysed cells: 71.4%).


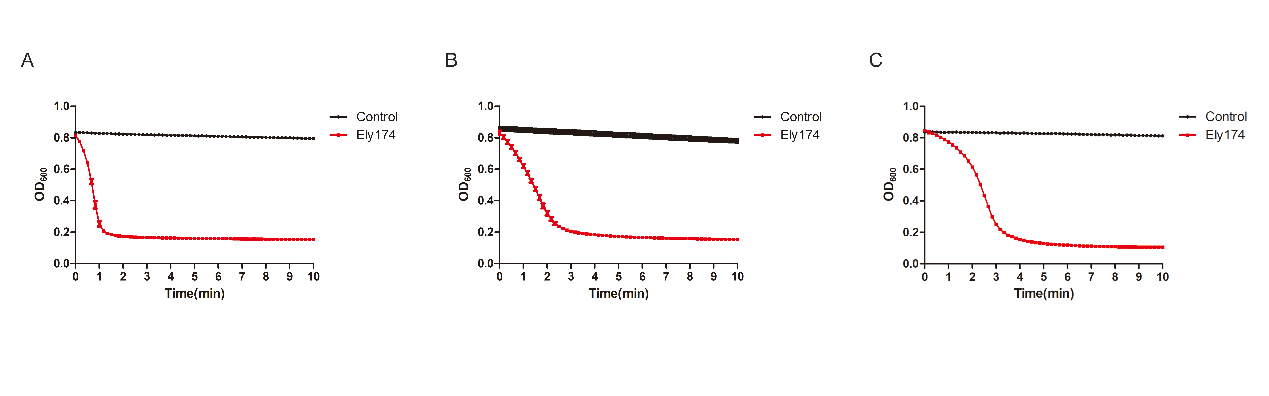


**FIG S3** Triton-pretreated *Flavobacterium facile* (A), *Chryseobacterium* *balustinum* (B), and *Flavobacterium* *psychrophilum* DSM3660 (C) were lysed by endolysin Ely174. Control: Tris-HCl buffer.


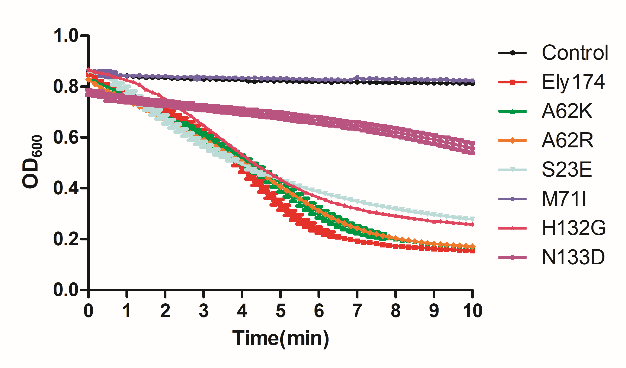


**FIG S4** Lytic activity of endolysin Ely174 and its variants. *F.psychrophilum* JC pretreated by Triton was used for detection. Control: Tris-HCl buffer.


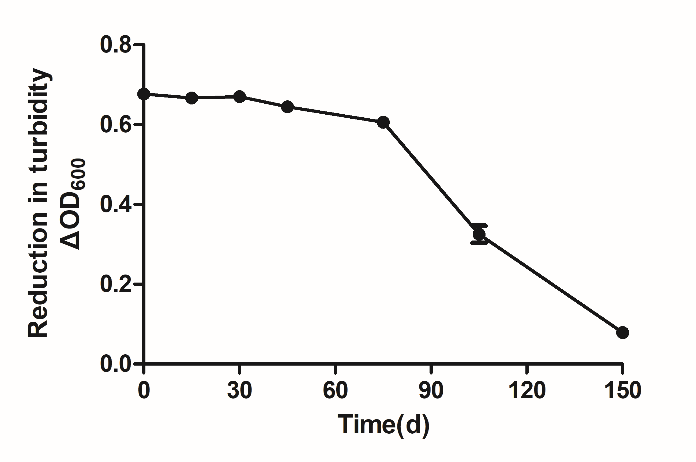


**FIG S5** Storage stability of endolysin Ely174 at 4℃.


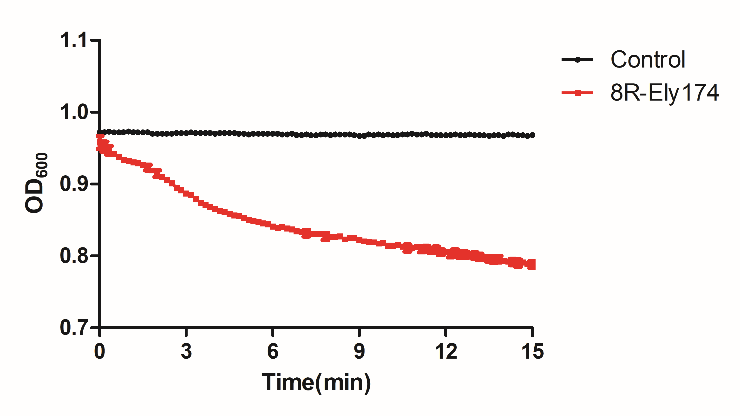


**FIG S6** The natural *F. psychrophilum* cells were lysed by engineered endolysin 8R-Ely174 (20 µg/mL). Control: Tris-HCl buffer.


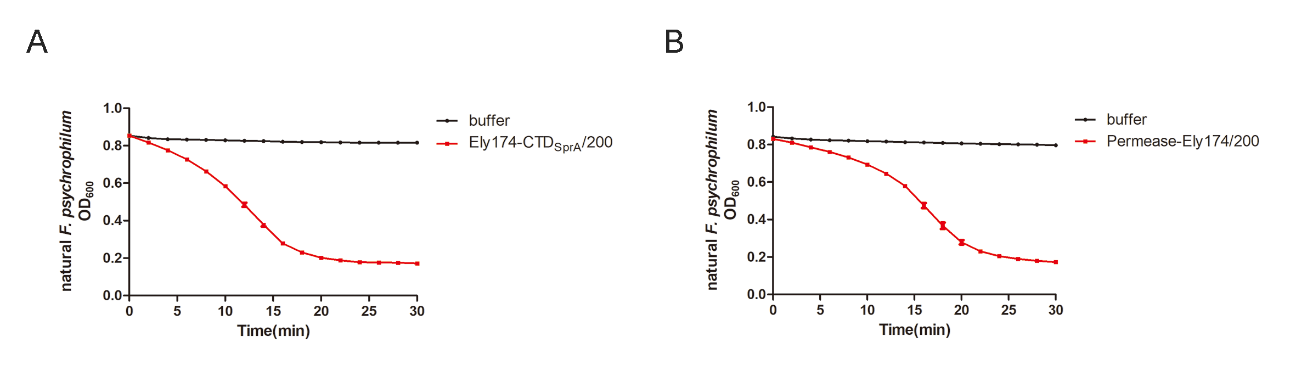


**FIG S7** The natural *F. psychrophilum* cells were lysed by engineered endolysin Ely174 -CTD_SprA_ and Permease-Ely174 (200 µg/mL).
